# Acute effects of unilateral sectioning the superior ovarian nerve of rats with unilateral ovariectomy on ovarian hormones (progesterone, testosterone and estradiol) levels vary during the estrous cycle

**DOI:** 10.1186/1477-7827-9-34

**Published:** 2011-03-18

**Authors:** Angélica Flores, Jacqueline Velasco, Alma I Gallegos, Fernando D Mendoza, Pamela M Everardo, María-Esther Cruz, Roberto Domínguez

**Affiliations:** 1 Biology of Reproduction Research Unit, FES Zaragoza UNAM, México City, México DF, México

## Abstract

The present study analyzed the participation of the left and right superior ovarian nerves (SON) in regulating progesterone, testosterone, and estradiol serum levels in unilaterally ovariectomized rats on each day of the estrous cycle. For this purpose, ovarian hormone concentrations in serum were measured in animals with either sham-surgery, unilateral ovariectomy (ULO), unilateral sectioning of the SON, or sectioning of the SON innervation of the *in situ *ovary in rats with ULO.

This investigation results show that the right and left ovaries have different capacities to maintain normal hormone levels, that such capacity varies during the estrous cycle, and that it depends on the integrity of the SON innervation. In rats with only one ovary, the effects of ovarian denervation on hormone levels varied according to which ovary remained *in situ*, the specific hormone, and the day of the estrous cycle when treatment was performed. Present results support the idea that the ovaries send and receive neural information that is processed in the central nervous system and we propose that this information participates in controlling the secretion of gonadotropins related to the regulation of ovarian functions.

## Background

Asymmetry in the ovaries' morphology, physiology, and regulatory structures is well established. Evidence suggesting that these asymmetries play an important functional role in regulating gonadal functions, and that the degree of asymmetry between gonads fluctuates along the estrous cycle, has been published [1]. The ovarian innervations play a role in regulating the ovulation process [2-5], in hormone secretion [6,7], and function as neural pathways that participate in modulating hypothalamic and non-hypothalamic centres that regulate the secretion of gonadotropins [1]. Furthermore, it has been proposed that the ovarian innervations modulate the reactivity of different ovarian compartments to gonadotropins effects [1,8].

The ovary receives its noradrenergic innervation via two main routes: the superior ovarian nerve (SON), which travels along the suspensory ligament; and the ovarian plexus nerves, which reach the ovary together with the main ovarian vessels [9-11]. Evidence that the ovary also receives vagal innervation has been published [12]. Aside from the classical neurotransmitters (noradrenaline (NA) and acetylcholine), several polypeptide neurotransmitters have been documented in the innervations arriving and leaving the ovaries [13-19].

According to Uchida et al [20], neural reflexes from the abdominal skin to the ovaries affect ovarian blood flow and the activity of the SON. The response level depended on whether the left or right abdominal afferent was stimulated, since stimulating the left abdomen produced a much stronger effect on the activity of the left ovarian sympathetic nerve than stimulating the right abdomen. The response of ovarian blood flow to abdominal stimulation is mediated as a reflex response via the ovarian sympathetic nerves, and the response is controlled via supra-spinal pathways and depends on the estrous cycle [21]

Niswender et al. [22] suggest that there are evidence indicating that ovarian blood flow is an important factor regulating the activity of gonadotropic hormones at the luteal cell level, and a secondary mechanism of action of LH may be to increase blood flow to the corpus luteum.

Ovarian and uterine arteries with anastomosis between them, provide arterial blood supply to the ovaries. Blood flow to the ovaries varies in magnitude and distribution throughout the estrous cycle [23-25], and the number and distribution of the follicular and luteal capillaries changes throughout the estrous cycle [26].

Most neurones originating from the SON fibers are located in the complex celiac-mesenteric ganglia (CSMG). The SON carries most of the catecholaminergic fibers innervating endocrine ovarian cells, which are distributed in the peri-follicular theca layer and are closely related to the theca internal cells [9,27]. In prepubertal rats, 24 and 72 hrs after unilateral or bilateral sectioning of the SON, the NA levels in the denervated ovary were lower than in untouched (control) and laparotomized animals [28].

Aside from the catecholaminergic innervation, the SON provides vasoactive intestinal peptide (VIP) [29] and nitric oxide (NO) [30] innervations to the ovaries. NO inhibits cytochrome P450 aromatase activity and the secretion of estradiol (E2) by granulosa cells in culture [31]. *In vitro *studies show that in the rat, the participation of neurotransmitters regulating the secretion of ovarian progesterone (P4) varies along the day of the estrous cycle. In diestrus-1 (D1), neuropeptide Y (NPY), NA and VIP inhibit P4 secretion by the ovaries, while on diestrus-2 (D2) these neurotransmitters stimulate P4 secretion. On D1 and D2, the effects of NA + VIP or NA + NPY on P4 secretion were higher than VIP or NPY alone [23]. In the rat, ovary denervation reduces the synthesis and secretion of P4 by inhibiting 3-betaHSD activity [32]. In the pig, sectioning of the plexus nerve and the SON led to lower plasma levels of LH, P4, androstenedione (A4), testosterone (T), estrone and estradiol-17beta. Further, a significant increase in the immuno-expression of cholesterol side-chain cleavage cytochrome P450 in follicles, as well as a decrease of 3-betaHSD, and in plasma levels of luteinizing hormone (LH), P4, A4, T, estrone and estrogen have been documented [33].

Unilateral ovariectomy (ULO) is a useful tool for studying the mechanisms involved in the asymmetric responses of the ovaries to neuroendocrine regulating signals [34-37]. The difference between the right and left ovaries' capacity to release oocytes seems to be related to the type and degree of the innervations in each gonad [1]. According to Klein and Burden [10], the number of neural fibers received by the right ovary is higher than in the left; while, Toth *et al.*[38] showed that the left ovary sends more neural information to the central nervous system (CNS) than the right ovary. In addition, the right and left ovaries show different ovulatory responses to surgical denervation, and these responses vary according to the day of the estrous cycle when surgery is performed [3,39].

Ovarian denervation by sectioning the vagus nerve has different effects on normal cyclic rats and ULO rats. In normal cyclic rats sectioning the left vagus nerve resulted in lower ovulation rate than in sham operated animals, while sectioning the right vagus nerve did not modify the ovulation rate. Sectioning the right or left vagus nerves to right-ULO rats (left ovary *in-situ*) reduces compensatory ovarian hypertrophy. In turn, sectioning the left vagus nerve induced different effects depending on which ovary remained *in-situ*. Left-side vagotomy performed to right ULO rats (left ovary *in-situ*) resulted in higher ovulation rates, compensatory ovarian hypertrophy, and number of ova shed; while the same procedure to left ULO rats (right ovary *in-situ*) resulted in a decrease of the same parameters [2,3]. In rats, the electrical stimulation to the ovarian plexus nerve or the SON produces a vasoconstriction of ovarian arterioles and a reduction of ovarian blood flow in rats [33]. The stimulation of the SON resulted in a significantly decrease of E2, while electrical stimulation of the ovarian plexus nerve did not modify it. This suggests that autonomic nerves that reach the ovary via the SON have an inhibitory role in the secretion of ovarian E2 [40].

Sensorial innervations also play a role in regulating ovarian functions. Sensorial denervation induced by capsaicin injection, systemic or into the ovarian bursa, diminished spontaneous ovulation and secretion of P4 and E2 [4]. Capsaicin treatment to ULO rats affect ovulation and the secretion of ovarian steroids depending on which ovary remained *in situ *and the day of the cycle when treatment was performed [41,42].

By comparing hormone levels in untouched (control) and ULO rats, this investigation studied the participation of the SON innervation in regulating hormone secretion by the left and right ovaries. The following hypotheses were assessed:

1) Since the innervations arising from the ovaries carry neural signals to the CNS, then, extirpating one ovary will produce acute changes in the neuroendocrine mechanisms regulating hormone secretion by the *in situ *ovary, and the type and magnitude of these changes would depend on which ovary (left or right) remains *in situ *as well as on which day of the estrous cycle surgery is performed.

2) Because the participation degree of the ovarian innervations on the modulation of hormone secretion seems to depend on the day of the estrous cycle, then, the acute effects of unilaterally sectioning the SON on P4, T and E2 serum levels will depend on the day of the cycle when denervation is performed.

3) Since after ULO the CNS no longer receives the neural information arising from the extirpated ovary, then, denervating the *in situ *ovary of animals with ULO, by sectioning the SON, will result in different hormone secretion changes than those resulting from sectioning the SON of animals with both ovaries *in situ*.

4) Since the neural regulation of ovarian functions seems to be asymmetric and to vary along the estrous cycle, then the changes in P4, T and E2 levels observed in animals with ULO will depend on the ovary remaining *in situ* and the day of the estrous cycle when ULO surgery, is performed.

5) Since acute bilateral ovariectomy affects ovarian steroid serum levels in different ways, then the effects of ULO, SON sectioning, and ULO + SON sectioning will differ according to the manipulated organ and the hormone studied.

## Methods

For this investigation, virgin adult female rats (195-225-g body weight) of the CIIZ-V strain from our own stock were used. The experiments were performed following the guidelines established by The Mexican Law of Animal Protection Guidelines Treatment. The Committee of the Facultad de Estudios Superiores Zaragoza approved the experimental protocols.

Animals were kept under controlled lighting conditions (lights on from 05:00 to 19:00 h), with free access to food (Purina S.A., Mexico) and tap water. Estrous cycles were monitored by daily vaginal smears; only rats showing at least two consecutive 4-day cycles were used in the experiment.

Rats were randomly allotted to one of the five experimental groups described below. Animals from different experimental groups were treated simultaneously and sacrificed one hour after surgery (14.00-14.15 h). All surgeries were performed in rats under ether anesthesia, using a ventral approach 13.00-13.15 hrs on each day of the estrous cycle. The animals woke up immediately after surgery.

### Experimental groups

The number in parenthesis indicates the number of animals in each group.

#### Control group_(N = 40)

Non-treated cyclic rats (ten animals on each day of the estrous cycle) were sacrificed between14:00 and14:15 h on diestrus 1 (D1), diestrus 2 (D2), proestrus (P) or estrous (E).

#### Sham surgery

An incision, affecting skin, muscle, and peritoneum, was performed 2 cm below the sternum [D1 (9), D2 (10), P (10) and E (10)]. The wound was subsequently sealed. No organs were extirpated or handled.

#### Unilateral ovariectomy (ULO)

A similar incision to that described for sham-surgery treatment was performed to extirpate the right ovary [D1 (9), D2 (9), P (10) and E (10)] or the left ovary [D1 (9), D2 (9), P (9) and E (9)]; the wound was subsequently sealed.

#### Unilateral sectioning of the superior ovarian nerve

A similar incision to that described for sham-surgery treatment was performed; the right [D1 (9), D2 (9), P (9) and E (8)] or left [D1 (10), D2 (10), P (10) and E (9)] ovary was exposed and the SON of the ovary was sectioned, as previously described by Chávez *et al. *[4]. The wound was subsequently sealed.

#### Unilateral section of the SON to ULO animals

The right [D1 (9), D2 (9), P (10) and E (10)] or left ovary [D1 (10), D2 (10), P (10) and E (9)] was extirpated and the SON of the *in situ *ovary was sectioned immediately after. The wound was subsequently sealed.

**Figure 1:**
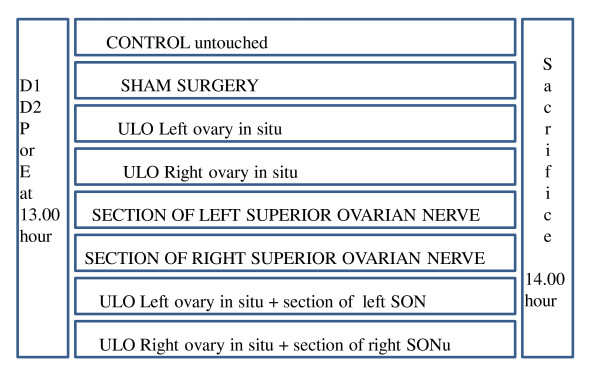
**The diagram shows a summary of the treatments**. ULO = unilateral ovariectomy. D1, D2, P or E = Days of the estrous cycle when surgeries were performed.

Figure 1 shows a summary of the treatments

### Autopsy procedures

Rats were sacrificed by decapitation; the blood of the trunk was collected, allowed to clot at room temperature for 30 minutes, and centrifuged at 3,000 rpm during 15 minutes. Serum was stored at -20°C, until P4, T and E2 concentrations were measured.

### Hormone assay

Concentrations of P4, T, and E2 in serum were measured using Radio-Immuno-Assay (RIA); with kits purchased from Diagnostic Products (Los Angeles, CA). Analytical results are expressed in ng/ml (P4) and pg/ml (T and E2). The intra- and inter-assay percent variation coefficients for P4 were 5.3 and 9.87; for T 5.6 and 8.7 and for E2 6.9 and 10.8, respectively. The detection limits of: P4 0.05 ng/ml to 40 ng/ml; correlation coefficient 0.9991; T 0.0020 ng/ml to 8.0 ng/ml, correlation coefficient 0.9851; E2 0.2680 pg/ml to 900.00 pg/ml; correlation coefficient 0.9960.

### Statistics

Data on hormonal concentrations in serum were analyzed using multivariate analysis of variance (MANOVA), followed by Turkey's test. Differences in serum hormone concentrations between two groups were analyzed using the Student's t-test. A probability value of less than 5% was considered significant.

## Results

### Effects of sham-surgery (Table 1)

**Table 1:** Mean ± SEM of progesterone (ng/ml), testosterone and estradiol (pg/ml) serum levels in control and sham treated animals during each day of the estrous cycle

GROUP	N	D1	N	D2	N	P	N	O
PROGESTERONE								
**Control**	10	24.0 ± 2.8	10	7.1 ± 1.2	10	14.1 ± 2.6	10	24.1 ± 2.4
**Sham**	9	63.5 ± 11.1*	10	33.0 ± 4.4*	10	28.7 ± 2.4*	10	26.3 ± 1.1
TESTOSTERONE								
**Control**	10	10.8 ± 2.0	10	73.5 ± 9.8	10	145.8 ± 18.2	10	<2.0
**Sham**	9	19.1 ± 8.6	10	103.4 ± 14.0*	10	285.8 ± 20.8*	10	76.5 ± 20.8*
ESTRADIOL								
**Control**	10	59.3 ± 4.6	10	38.9 ± 2.8	10	130.9 ± 13.1	10	29.1 ± 2.4
**Sham**	9	46.6 ± 5.6	10	38.6 ± 2.8	10	149.7 ± 11.3	10	30.2 ± 2.6

* p < 0.05 vs. Control (Student's t test)

Compared to the control group, sham surgery on D1, D2 and P resulted in higher P4 concentrations, while sham surgery performed on D2, P or E resulted in higher T concentrations. No changes in E2 serum concentrations were observed. Based on these results, the effects of experimental surgeries were compared to their corresponding sham surgery group.

### Effects of ULO (Table 2)

**Table 2:** Mean ± SEM of progesterone (ng/ml), testosterone and estradiol (pg/ml) serum levels in unilateral ovariectomized rats (ovary in situ)

GROUP	N	D1	N	D2	N	P	N	O
PROGESTERONE								
**Sham**	9	63.5 ± 11.1	10	33.0 ± 4.4	10	28.7 ± 2.4	10	26.3 ± 1.1
**R-OVARY**	10	78.1 ± 11.6	10	38.5 ± 3.0	10	25.8 ± 2.1	8	35.2 ± 4.0
**L-OVARY**	9	39.2 ± 3.1*	9	32.3 ± 4.3	8	28.7 ± 3.0	10	68.3 ± 11.3*
TESTOSTERONE								
**Sham**	9	19.1 ± 8.6	10	103.4 ± 14.0	10	285.8 ± 20.8	10	76.5 ± 20.8
**R-OVARY**	10	13.3 ± 3.6	10	128.2 ± 13.7	10	177.3 ± 24.7*	8	26.7 ± 8.9*
**L-OVARY**	9	36.5 ± 15.4	9	77.8 ± 14.6	8	225.8 ± 15.4	10	68.5 ± 11.8
ESTRADIOL								
**Sham**	9	46.6 ± 5.6	10	38.6 ± 2.8	10	149.7 ± 11.3	10	30.2 ± 2.6
**R-OVARY**	10	25.7 ± 4.2*	10	31.0 ± 2.9	10	120.4 ± 12.4	8	31.2 ± 2.9
**L-OVARY**	9	36.8 ± 4.2	9	35.5 ± 1.3	8	71.1 ± 10.9*^&^	10	26.4 ± 2.0

Performed at 13.00 h of diestrus 1, diestrus 2, proestrus or estrus, sacrificed one hour after treatment

*p < 0.05 vs. Control (ANOVA followed by Tukey's test); ^&^p < 0.05 vs. R-Ovary (ANOVA followed by Tukey's test)

#### Effects on P4 serum levels

Compared to sham-surgery animals, left ULO (right ovary *in-situ*) did not modify P4 serum levels; regardless of the day treatment was performed. Right ULO (left ovary *in-situ*) performed on D1 resulted in lower P4 levels, while the same treatment performed on E resulted in higher P4 concentrations.

#### Effects on T serum levels

Compared to sham-surgery animals, left ULO (right ovary *in-situ*) performed on P or E resulted in lower T. Compared to sham-surgery animals, right ULO (left ovary *in-situ*) did not modify hormone serum levels, regardless of the day of the estrous cycle when surgery was performed. Right ULO (left ovary *in-situ*) on E resulted in higher T levels than in animals with left ULO (right ovary *in situ*).

#### Effects on E serum levels

Left ULO (right ovary *in-situ*) on D1 as well as right ULO (left ovary *in-situ*) on P resulted in lower E2 serum levels. For animals treated on P, right ULO (left ovary *in situ*) resulted in lower E2 concentrations than in rats with left ULO (right ovary *in situ*) treatment.

**Figure 2:**
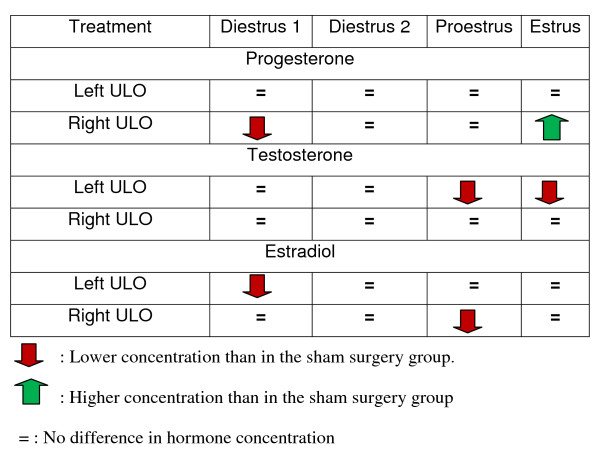
**Summary of ULO treatment effects**. P4, T and E in serum compared to hormone concentrations in animals with sham-surgery treatment.

Figure 2 shows a summary of the effects of ULO on P4, T and E serum levels

### Effects of Sectioning the SON of rats with both ovaries *in situ *(Table 3)

**Table 3:** Mean ± SEM of progesterone (ng/ml), testosterone and estradiol (pg/ml) serum levels in rats with unilateral sectioning of the SON

GROUP	N	D1	N	D2	N	P	N	O
PROGESTERONE								
Sham	9	63.5 ± 11.1	10	103.4 ± 14.0	10	149.7 ± 11.3	10	26.3 ± 1.1
R-SON	10	36.3 ± 2.2*	8	115.1 ± 15.7	9	122.9 ± 12.1	8	40.2 ± 4.1*
L-SON	10	51.7 ± 11.0	8	62.2 ± 13.8*#	7	119.1 ± 11.2	8	71.2 ± 6.7*#
TESTOSTERONE								
Sham	9	19.1 ± 8.6	9	103.4 ± 14.0	10	285.8 ± 28.0	10	76.5 ± 20.8
R-SON	10	20.3 ± 1.9	10	115.1 ± 15.7	9	194.7 ± 22.1*	8	111.3 ± 30. 1
L-SON	10	51.0 ± 8.4*	10	62.2 ± 13.8*#	7	306.2 ± 22.2#	8	56.7 ± 10.9
ESTRADIOL								
Sham	9	46.6 ± 5.6	9	38.6 ± 2.8	10	149.7 ± 11.3	10	30.2 ± 2.6
R-SON	10	40.9 ± 4.5	10	42.7 ± 5.7	9	122.9 ± 12.1	8	48.9 ± 5.9
L-SON	10	43.1 ± 5.9	10	49.5 ± 3.2	7	119.1 ± 11.2	8	62.0 ± 12.1

Performed at 13.00 h of diestrus 1, diestrus 2, proestrus or estrus, sacrificed one hour after treatment

*p < 0.05 vs. Control: # p < 0.05 vs. R-SON (ANOVA followed by Tukey's test]

Depending on the day of treatment, sectioning the SON of rats with both ovaries *in situ *had different effects on the concentrations of ovarian hormones in serum.

#### Effects on P4 serum levels

Compared to sham-operated animals, sectioning the right SON on D1 or E resulted in higher P4 levels. In rats treated on D2, sectioning the right SON resulted in higher P4 levels than sectioning the left SON; while sectioning the left SON on E resulted in higher P4 levels than sectioning the right SON and in the sham-surgery group.

#### Effects on T serum levels

Sectioning the left SON on D1 resulted in higher T levels than sham-surgery or sectioning the right SON treatment. In turn, sectioning the left SON on D2 resulted in lower T levels than sham-surgery or sectioning the right SON treatment, while sectioning the right SON on P resulted in lower T levels than sham-surgery or sectioning the left SON treatment.

Regardless of the day surgery was performed, sectioning the left or right SON did not modify E2 concentrations in serum.

**Figure 3:**
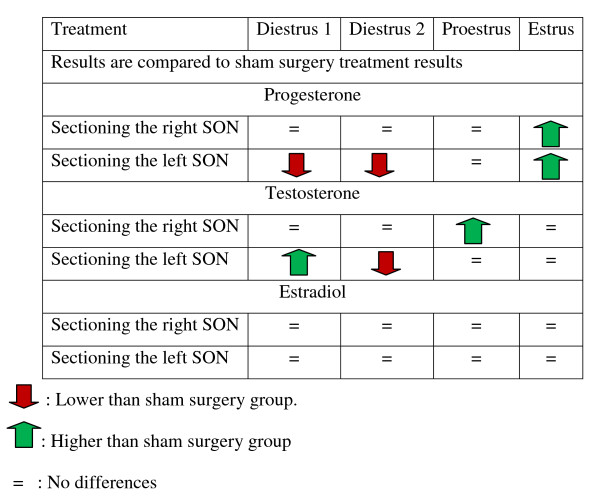
**Summary of the effects of sectioning the SON on P4, T and E serum levels**.

Figure 3 shows a summary of the effects of sectioning the SON on P4, T and E serum levels

### Effects of Sectioning the SON in rats with ULO

#### Effects on P4 serum levels

**Figure 4:**
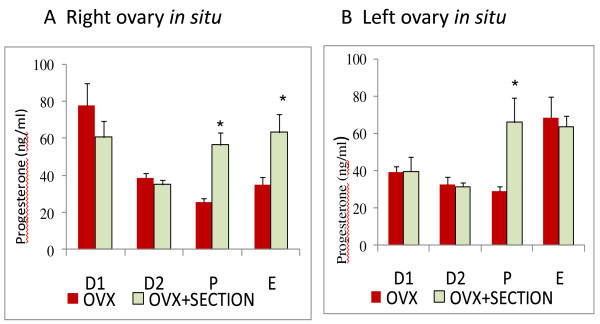
**Progesterone serum levels**. Means ± SEM of progesterone serum levels (ng/ml) in rats with unilateral ovariectomy (OVX) and unilateral ovariectomy followed the section of the superior ovarian nerve (OVX + SECTION) of the *in situ *ovary (A - right in situ ovary; B - left in situ ovary). * p < 0.05 vs. OVX (Student's test).

Sectioning the right SON of animals with left ULO (right ovary in situ) on P or E resulted in higher P4 serum levels than in rats with left ULO (Figure 4A). In rats with right ULO (left ovary *in situ*) treatment, sectioning the left SON on P resulted in higher P4 levels (Figure 4B).

#### Effects on T serum levels

**Figure 5:**
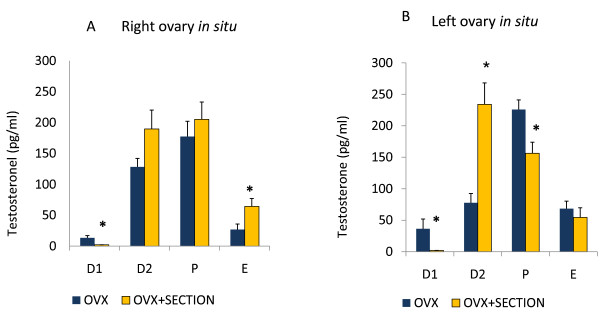
**Testosterone serum levels**. Means ± SEM of testosterone serum levels (pg/ml) in rats with unilateral ovariectomy (OVX) and unilateral ovariectomy followed the section of the superior ovarian nerve (OVX + SECTION) of the in situ ovary (A - right in situ ovary; B - left in situ ovary). * p < 0.05 vs. OVX (Student's test).

In rats with left ULO (right ovary *in situ*) treatment, sectioning the right SON on D1 resulted in lower T levels than in ULO treated rats; while the same treatment performed on E resulted in higher T levels (Figure 5A). On D1 or P, sectioning the left SON to ULO rats resulted in lower T levels than in rats with the left ovary *in situ*, while the same treatment performed on D2 resulted in higher T levels than in ULO rats (Figure 5B).

#### Effects on E2 serum levels

**Figure 6:**
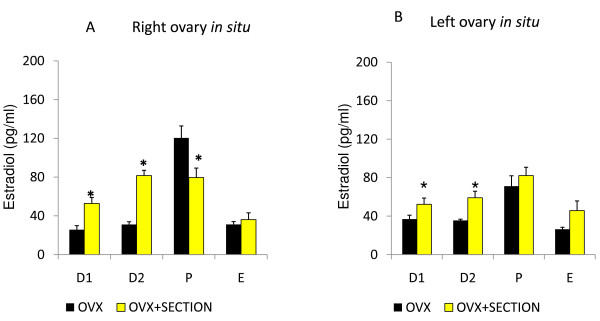
**Estradiol serum levels**. Means ± SEM of estradiol serum levels (pg/ml) in rats with unilateral ovariectomy (OVX) and unilateral ovariectomy followed the section of the superior ovarian nerve (OVX + SECTION) of the in situ ovary (A - right in situ ovary; B - left in situ ovary). *p < 0.05 vs. OVX (Student's test).

Sectioning the right SON of rats with left-ULO (right ovary *in situ*) on D1 or D2 resulted in higher E2 levels; while the same treatment on P resulted in lower E2 levels (Figure 6A). On the other, sectioning the left SON of rats with right-ULO (left ovary *in situ*) on D1 or D2 resulted in higher E2 levels than in rats with ULO (Figure 6B).

### Comparative effects of unilaterally sectioning the SON of rats with both ovaries and ULO rats (figure 7)

**Figure 7:**
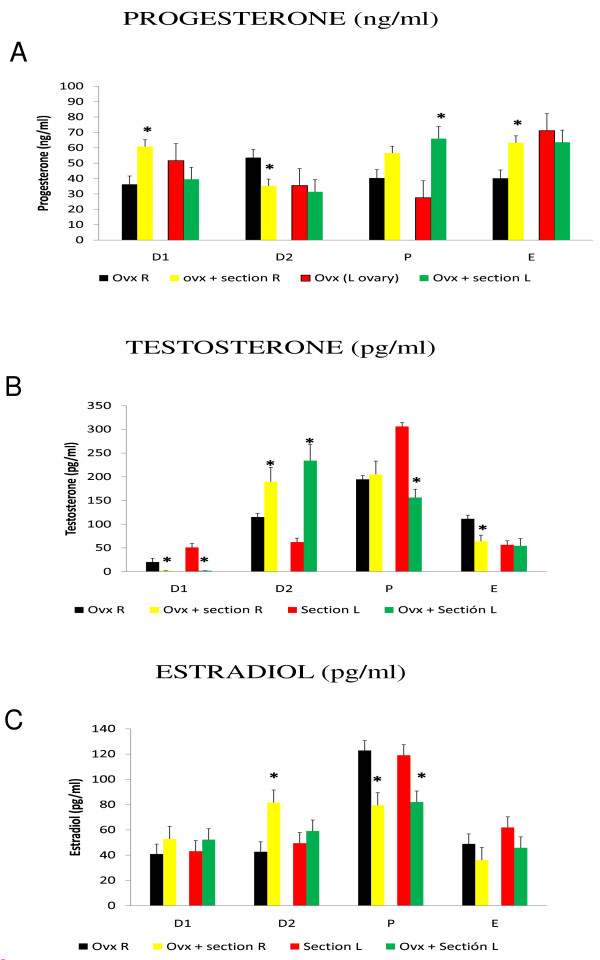
**Comparative effects on progesterone, testosterone and estradiol**. Means ± SEM of comparative effects of unilateral section of the SON on progesterone, testosterone and estradiol between rats with unilateral section of the SON and rats with unilateral ovariectomy (OVX) followed by the unilateral section of the SON. * p < 0.05 vs. section (Student's test).

#### Effects on P4 serum levels

In rats treated on D1 or E, P4 levels were higher in rats with ULO + sectioning the right SON than in rats with both ovaries and sectioning of the right SON treatment. Rats treated on D2 had an inverse response.

#### Effects on T serum levels

In rats treated with ULO + sectioning the SON on D1, T levels were lower than in rats with both ovaries and sectioningof the SON (right or left) treatment. An inverse result occurred in rats treated on D2. Rats treated with ULO + sectioning the left SON on P had lower T levels than rats with both ovaries and sectioning of the left SON. In rats treated on E, rats with ULO + sectioning the right SON had lower T levels than rats with both ovaries and section of the right SON.

#### Effects on E2 serum levels

Rats with ULO + sectioning the right ovary treatment on D2, had higher E2 levels than rats with both ovaries and section of the right or left SON. Rats with ULO + sectioning the SON on P had lower E2 levels than rats with both ovaries and section of the right or left SON.

**Figure 8:**
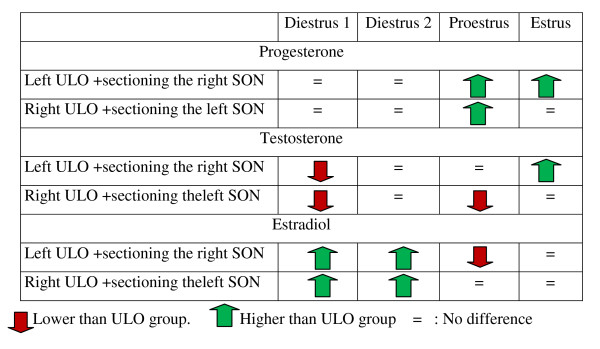
**Summary of the effects of sectioning the SON to ULO rats on P4, T and E serum levels**. Results were compared to the respective ULO treatment group.

Figure 8 shows a summary of the effects of sectioning the SON to ULO rats on P4, T and E serum levels. Results were compared to the respective ULO treatment group.

## Discussion

The results presented herein support the hypotheses that secretion of ovarian steroid hormones is asymmetric and depend on the neural information arriving to the ovaries through the SON.

The results also support the hypothesis that the secretion of steroid hormones levels varies through the estrous cycle. The results suggest that the acute extirpation of one ovary modifies the mechanisms regulating hormone secretion and that these modifications depend on the extirpated ovary and the day of the cycle when surgery is performed.

Kawakami et al., [43,44] showed that electric stimulation of the ventromedial hypothalamus and of the medio-basal prechiasmathic area in hypophysectomized and adrenalectomized rats provoked the release of P4 and E2 with no modifications in the levels of gonadotropins, or ovarian blood flow, and GnRH [45] suggesting a direct neural control of the ovarian steroidogenesis. In the pre-pubertal rat the differences on P4, T and E2 levels induced by right- or left-ULO did not correlate with changes in FSH or LH concentrations, suggesting that the acute effects of unilateral ovariectomy on P4, T and E2 secretion by the ovaries does not depend on gonadotropin signals [46].

Noradrenaline and vasoactive intestinal peptide (VIP) stimulate the ovarian release of P4, while GnRH and gamma aminobutyric acid (GABA) play an inhibitory role. Some of these neurotransmitters are also present in the SON and the coeliac ganglion [39]. *In vitro *studies by Garraza et al. [29] show that NPY, VIP or SP applied directly on the ovaries obtained from rats on D1 inhibit the secretion of P4, while the same treatment on ovaries from rats on D2 stimulates P4 secretion. The participation of the ovaries and adrenals in maintaining normal P4 levels vary during the estrous cycle. There is evidence indicating the ovaries release more P4 on D1 than the adrenals; while on D2, P and E, the main source of P4 are the adrenals [47]. In the present study, by comparison with untouched control rats, the increase of P4 levels in sham-surgery rats treated on D1 or D2 was higher than those observed in ether-anaesthetised rats (164% vs. 20.6%; 237% vs. 66.2%) [34,35] suggesting that the abdominal skin stimulation play a stimulatory role on P4 release during D1 and D2. A similar effect was observed with T levels in sham-surgery rats treated on D2 or E. The neural connections between the abdominal skin and the ovaries proposed by Uchida et al [20] would be the neural path used.

Present results show that removing the right ovary (left ovary *in situ*) on D1 resulted in lower P4 level, but removing the left ovary (right ovary *in situ*) results in hormone levels similar to animals with sham surgery treatment. The results suggest that the increase in P4 levels observed in animals with sham surgery treatment depend on the secretion activity by the right ovary.

Since sectioning the left SON on D1 or D2 resulted in lower P4 levels than in sham surgery treated rats, we propose that the neural reflex elicited by the sham surgery arrives to the left ovary through the left SON. The decrease in P4 levels could be explained by the decrease in ovarian NA and VIP quantities, since the unilateral or bilateral sectioning of the SON results in an acute decrease in NA levels [17,33], and both neurotransmitters stimulate ovarian P4 secretion [48].

As indicated by the present results, rats with right-ULO (left ovary *in situ*) on E showed higher P4 levels than animals in the sham-surgery treatment group. We suppose that the increase in P4 secretion originates in the adrenals. Since the noradrenergic nerves arriving to the ovaries and the adrenals originate at the CSMG [49], it is possible that some kind of neural information arising from the left ovary is carried to the CSMG resulting in the stimulation of the nerves innervating the adrenals. Since sectioning the left or right SON also resulted in higher P4 levels, we suppose that on the day of E the neural information carried by both SONs regulates P4 secretion in an inhibitory way, which does not include NA and VIP as neurotransmitters.

Removing the left ovary modified the way P4 secretion is regulated by the right ovary; since sectioning the right SON on P or E resulted in higher P4 levels than in ULO rats with the right ovary in situ. A similar effect occurred in rats with the left ovary *in situ *with section of the left SON at P.

According to Odell and Parker [50], the major adrenal androgens are dehydroepiandrosterone (DHEA), dehydroepiandrosterone sulphate (DHEAS), and A4; which are converted into androgen and estrogen by steroidogenic enzymes in the peripheral tissues [51]. In the rat, T secretion by the adrenals is limited [52]; and thus, the changes in hormone levels observed in response to ULO or denervation must be explained by modifications in the ovarian capacity to secrete T. In rats with ULO treatment the SON plays a stimulatory role in secreting T by the left ovary, while for the right ovary, the SON plays a stimulatory on D1 and an inhibitory role on E. Such difference may result from changes in the sensitivity of the ovary to the effects of LH and/or by the stimulation of P450 aromatase activity. On D1, ULO rats with a denervated ovary had lower T levels and higher E2 levels than rats with just ULO treatment.

At the day of estrus, the right SON plays an inhibitory role in ovarian E2 secretion [40]. ULO acute effects on E2 levels were observed on D1 for the right ovary and on P for the left ovary. For the right ovary the changes in E2 secretion depend on the innervations provided by the SON, but it does not for the left ovary, suggesting that the left and right ovaries have different kinds of regulation.

*In vitro *studies show that the ovaries'ability to secrete hormones changes with the presence or absence of a diverse group of neurotransmitters [21,53-55]. Then, the acute effects of ULO or sectioning the SON seem to influence the activity and/or the expression of enzymes participating in the synthesis of steroid hormones produced by the ovaries.

In ULO rats, sectioning the SON of the *in situ *ovary results in a different response than that observed in animals with both ovaries; suggesting that the effects of ULO result from the modifications in ovarian hormone levels, and the alterations on the neural information arising from the extirpated organ [1].

The variations in the asymmetrical performance by the right and left ovaries during the estrous cycle could be related to changes in the neural information received by the ovaries from the CSMG. Morán et al [56] showed that the neural connections between the CSMGs and the right and left ovaries show a mirror-image vary along the estrous cycle, and that the left ovary, but not the right one, has connections with both CSMGs. Furthermore, the SON is the main neural pathway connecting the ovaries to the CSMG [48].

Mortality for neurological or mental diseases is higher in women who underwent bilateral oophorectomy before age 45 years compared with referent women and it is not attenuated by estrogen treatment from the time of oophorectomy [57].

In women of reproductive age, the most common endocrinopathy is polycystic ovary syndrome (PCOS). There is evidence that PCOS is associated with increased sympathetic nerve activity, since repeated low-frequency electro-acupuncture treatment induced regular ovulation in women with PCOS [58], inhibited hyperactivity in the sympathetic nervous system [59] and improved hyperandrogenism [58,60]. Bilateral sectioning of the SON to rats with PCOS, induced by estradiol valerate, restored ovulation [61], while unilateral sectioning of the SON restored ovulation in the innervated ovary [62]. The neural reflexes induced from the abdominal skin to the ovaries affect ovarian blood flow and SON activity [20] and low-frecuency electro-acupuncture treatment is achieved by acting on the wall of the abdomen. Then, it is possible that their effects could be related with changes in the SON activity. Such possibility is based in present results (acute changes in P4, T and E2 levels induced by ovarian denervation and elimination of one of the neural communication between the ovary and the CNS) and Kagitani et al.[40] results showing that SON stimulation results in a decrease in E2 secretion

Taken together, present and previous results indicate that the mechanism regulating steroid hormones secretion by the ovaries is different for each hormone and for each ovary. Endocrine signals originate from, and arrive to, the ovaries; the ovaries receive and send neural information that is processed in the CNS and participate in regulating the secretion of gonadotropins related to the regulation of ovarian functions.

## Competing interests

The authors declare that they have no competing interests.

## Authors' contributions

AF and RD conceived and designed the study. JV, AIG, FDM and PME performed the surgeries and hormone measurements. MEC participated in the analysis and discussion of the results. RD prepared the initial draft of the manuscript. All co-authors provided inputs during final manuscript preparation. All authors read and approved the final manuscript.
